# In Vivo Microbial Coevolution Favors Host Protection and Plastic Downregulation of Immunity

**DOI:** 10.1093/molbev/msaa292

**Published:** 2020-11-12

**Authors:** Suzanne A Ford, Kayla C King

**Affiliations:** Department of Zoology, University of Oxford, Oxford, United Kingdom

**Keywords:** protective microbes, host–pathogen interactions, host–symbiont interactions, immune response, experimental evolution, microbial coevolution

## Abstract

Microbiota can protect their hosts from infection. The short timescales in which microbes can evolve presents the possibility that “protective microbes” can take-over from the immune system of longer-lived hosts in the coevolutionary race against pathogens. Here, we found that coevolution between a protective bacterium (*Enterococcus faecalis*) and a virulent pathogen (*Staphylococcus aureus*) within an animal population (*Caenorhabditis elegan*s) resulted in more disease suppression than when the protective bacterium adapted to uninfected hosts. At the same time, more protective *E. faecalis* populations became costlier to harbor and altered the expression of 134 host genes. Many of these genes appear to be related to the mechanism of protection, reactive oxygen species production. Crucially, more protective *E. faecalis* populations downregulated a key immune gene, , known to be effective against *S. aureus* infection*.* These results suggest that a microbial line of defense is favored by microbial coevolution and may cause hosts to plastically divest of their own immunity.

## Introduction

Organisms have repeatedly evolved methods to defend themselves against pathogen attack, yet the host microbiota also acts to prevent infection in plants and animals (Mejía et al. 2002; Martín-Platero et al. 2006; Ford and King 2016). Importantly, microbes can evolve quickly due to short generation times and strategies that favor innovation and variation (Hall et al. 2017). This potential for rapid evolution presents the possibility that components of the microbiome could adapt to defend against pathogen infection within a host’s lifetime (King et al. 2016; Ashby and King 2017) and even coevolve against pathogens (Kwiatkowski et al. 2012; Ford et al. 2017; Vorburger and Perlman 2018). 

Coevolution between microbial species is likely ongoing within long-lived hosts. The existence of these interactions is suggested by patterns of protective microbe–pathogen specificity in natural systems (Koch and Schmid-Hempel 2012; Rouchet and Vorburger 2012; Vorburger and Perlman 2018) and in theoretical work (Kwiatkowski et al. 2012). Microbial coevolution has been predicted to drive an increase in protective ability and also the cost to hosts of possessing protective microbes (Nelson and May 2020). Many natural defensive symbioses are costly to the host constitutively or in a context-dependent way, such as in the absence of enemy attack or in different environments (Oliver et al. 2014). The costs conferred by microbial symbionts can be a major determinant of their ability to spread in a host population (Oliver et al. 2008). Moreover, with competition between microbial species a dominant interaction within the host microbiome (Granato et al. 2019), over coevolutionary time enhanced host resource exploitation—and thus virulence or cost—could be favored (Frank 1996; West and Buckling 2003). Coevolutionary interactions could therefore alter the impact of resident microbes on the host, including their associated costs and protective effects (Nelson and May 2020).

If the net benefits of microbe-mediated protection are high enough to outweigh the costs, hosts might be freed from responding to pathogens themselves. Whether protective microbes could “take-over” defenses might be determined by whether the host itself is involved in the protective process. In some symbioses, the protective microbial species primes a component of the immune system (Mejía et al. 2002; Weiss et al. 2012). However, it is also frequently observed in nature for protective microbes to suppress pathogens via resource competition (Lindsey et al. 2018) or production of toxic molecules (Degnan and Moran 2008; Kroiss et al. 2010; Pan et al. 2012). Such direct suppression of pathogens by protective microbial species, may make parts of the host immune response during infection redundant. It is unclear whether hosts can plastically depend on their microbiota for antipathogen defenses. Theory (Vorburger and Perlman 2018; Metcalf and Koskella 2019) and empirical work (Martinez et al. 2016) suggest that microbe-mediated protection could select for reduced host investment in defense over evolutionary time. The loss of key immune genes in symbiont-colonized host species (Gerardo et al. 2010; Parker et al. 2011; Kaltenpoth and Engl 2014) is also a pattern suggestive of divestment of host-based immunity in defensive symbioses.

**Fig. 1.**
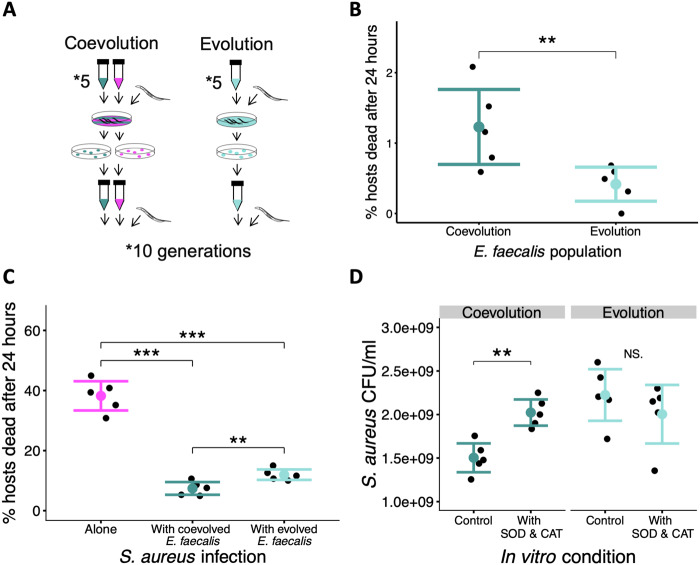
In vivo coevolution with pathogens favors costlier, but more protective microbes. (*A*) Within nonevolving *Caenorhabditis elegans* hosts, *Enterococcus faecalis* populations were either coevolved with *Staphylococcus aureus* or were evolved on their own. This procedure was continued for ten passages (see Materials and Methods for full protocol). (*B*) Host mortality (cost) due to colonization by coevolved/evolved *E. faecalis*. Binomial GLM: df = 1, *P* = 0.0062. (*C*) Host mortality following exposure to *S. aureus* exposure alone (magenta) or with either coevolved or evolved *E. faecalis* (dark or light turquoise, respectively). Quasibinomial GLM: *F* = 122.34, df = 2, *P* = 1.044e-08. Post hoc Tukey: *S. aureus* alone versus with coevolved *E. faecalis*: *P* < 0.001; *S. aureus* alone versus with evolved *E. faecalis*: *P* < 0.001. Coevolved *E. faecalis* versus with evolved *E. faecalis*: *P* = 0.005. (*D*) In vitro population size of *S. aureus* in colony-forming units per ml (CFU/ml) after coculture with either coevolved or evolved *E. faecalis*. CFUs/ml were also counted upon the addition of superoxide dismutase and catalase enzymes which remove superoxide and hydrogen peroxide from the media. *t*-Test, coevolution: control versus SOD&CAT, *t* = −4.6, df = 8, FDR-adjusted *P* = 0.003; evolution: control versus SOD&CAT, *t* = 0.98, df = 8, FDR-adjusted *P* = 0.35. All experiments were repeated twice and results were combined. Each treatment was replicated five times. *P < 0.05, **P < 0.01, ***P < 0.001. Bar plots show ±2SE.

We experimentally copassaged a protective bacterium (*Enterococcus faecalis*) and pathogen (*Staphyloccocus aureus*) within nonevolving nematode (*Caenorhabditis elegans*) populations to track changes in the cost (protective bacterium-induced host mortality) and benefit (reduction in pathogen-induced mortality) of the protective microbe to the host, along with the host’s transcriptional response. This nematode is well established for investigating host–microbe associations (Clark and Hodgkin 2014; Petersen et al. 2015). *Enterococcus faecalis* is naturally protective in a variety of animals (Heikkilä and Saris 2003; Martín-Platero et al. 2006), and although *C. elegans* likely encounter *Staphylococcus* species in their natural habitat (Montalvo-Katz et al. 2013; Rossouw and Korsten 2017), specific interactions with *S. aureus* here are considered novel. These microbial species can undergo antagonistic coevolution in vivo (Ford et al. 2017). We have previously shown that within nematodes, *E. faecalis* adapts to interactions with *S. aureus* by increasing the production of reactive oxygen species (ROS) that suppress pathogen growth (King et al. 2016). *Enterococcus faecalis* can also exploit the iron-binding siderophores produced by *S. aureus* (Ford et al. 2016) favoring the evolution of reduced *S. aureus* virulence through decreased siderophore production (Ford et al. 2016). In this study, we tested whether five independent *E. faecalis* populations that coevolved in vivo with *S. aureus* for ten host generations (isolates from Ford et al. 2016, 2017) demonstrated evolutionary changes in protective ability and cost relative to *E. faecalis* populations that evolved alone (fig. 1*A*; see Materials and Methods).

## Results and Discussion

### Coevolution Increased *E. faecalis* Protective Ability and Cost

Our results were consistent with in vivo microbial coevolution favoring more protective, but also costlier, protective microbes. We measured host mortality after 24 h of *E. faecalis* monocolonization and found the coevolved bacterium was significantly costlier relative to the evolved (fig. 1*B*, binomial GLM: df = 1, *P* = 0.0062). Although significant, the mortality caused by coevolved *E. faecalis* remained relatively low (1% mortality in the host population after 24 h). We cannot rule out that host mortality following *E. faecalis* colonization could have further increased linearly given more evolutionary time. It is also likely there are sublethal costs to *E. faecalis* colonization that we did not measure. In natural host-protective microbe relationships, constitutive or context-dependent costs measured are often sublethal and can cause reductions in host fitness (Oliver et al. 2014).

We then compared the protective ability of *E. faecalis* across treatments against a common, ancestral pathogen stock (see Materials and Methods). Although we found that *E. faecalis* colonization reduced pathogen-induced mortality in both treatments (fig. 1*C*, Quasibinomial GLM: *F* = 122.34, df = 2, *P* = 1.044e-08; post hoc Tukey: *S. aureus* alone vs. with coevolved *E. faecalis*: *P* < 0.001; *S. aureus* alone vs. with evolved *E. faecalis*: *P* < 0.001), coevolved *E. faecalis* was more protective than populations evolved as sole colonizers of the nematode gut (post hoc Tukey: with coevolved *E. faecalis* vs. with evolved *E. faecalis*: *P* = 0.005). This cost–benefit trade-off for the host is reflected in many natural defensive interactions (Polin et al. 2014; Martinez et al. 2015; for exception see Cayetano et al. 2015).

### Coevolution Increased *E. faecalis*-Mediated ROS Production

Our previous work showed that ROS production by *E. faecalis* contributes to host-protection and can evolve rapidly (King et al. 2016). To test for differences in ROS production between treatments, we measured *S. aureus* growth in liquid coculture with *E. faecalis*, in the presence and absence of enzymes that remove ROS, superoxide dismutase (SOD), and catalase (CAT, see Materials and Methods). SOD converts superoxide to hydrogen peroxide, whereas CAT converts hydrogen peroxide to water and oxygen. We found that only coevolved *E. faecalis* produced sufficient ROS to inhibit *S. aureus* growth (fig. 1*D*, *t*-test, Coevolution: control vs. SOD&CAT, *t*=−4.6, df = 8, FDR-adjusted *P* = 0.003; Evolution: control vs. SOD&CAT, *t* = 0.98, df = 8, FDR-adjusted *P* = 0.35). Consistent with this, we found numerous differences between our evolved *E. faecalis* populations at single nucleotide polymorphisms (SNPs) in genes linked to ROS production (supplementary table 1, Supplementary Material online). These data were collected by sequencing 40 pooled clones from each population. Supplementary table 1, Supplementary Material online, lists all the SNPs observed at frequencies higher than a minimum noise threshold of 15% per population. In the right-most column, we have annotated SNPs that appear to link to ROS. SNPs linked to ROS included ones that were within or next to genes involved in energy metabolism, flavin-containing oxidoreductases known to lead to ROS production (Messner and Imlay 2002), ROS scavenging, and the production and transport of xanthine which can be oxidized to produce ROS (Huycke et al. 2002). 

### Increased Protection Stimulates Iron and ROS Homeostasis in Infected Hosts

**Fig. 2.**
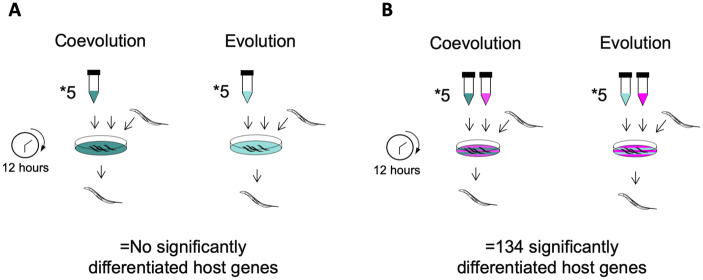
Experimental design measuring the effect of *Enterococcus faecalis* evolutionary treatment on *Caenorhabditis elegans* transcription in the absence and presence of *Staphylococcus aureus* infection. (*A*) *Caenorhabditis elegans* hosts were exposed to either the coevolved or evolved *E. faecalis* populations. After 12 h, worm RNA was extracted for gene expression analysis, and we found no significantly differentiated host genes. (*B*) *Caenorhabditis elegans* hosts were exposed to ancestral *S. aureus* alongside either the coevolved or evolved *E. faecalis* populations. After 12 h, worm RNA was extracted, and we found 134 genes that were differentially expressed between the treatments.

We tested whether increased microbe-mediated protection corresponded with reduced host immune gene expression during pathogen attack. First, we examined whether there were any differences in host transcription in response to colonization by coevolved and evolved *E. faecalis* populations in the absence of the pathogen (fig. 2*A*, see Materials and Methods). We did not find any genes that were significantly differentially regulated. This indicates that differences in protective ability or cost do not affect host transcription in uninfected hosts. Second, we examined whether there were differences in host transcription during colonization by the *E. faecalis* populations in the presence of the pathogen (fig. 2*B*, see Materials and Methods). We found that coevolved *E. faecalis* significantly altered the expression of 134 *C. elegans* genes relative to evolved *E. faecalis* within infected hosts (supplementary file 1, Supplementary Material online). We performed a GO-term enrichment analysis (see Materials and Methods) and found that these genes were significantly enriched for molecular functions that point to ROS production and iron metabolism, including: oxidoreductase activity (GO: 0016491, FDR-adjusted *P* = 1.5E-06), iron ion binding (GO: 0005506, FDR-adjusted *P* = 0.0001), heme binding (GO: 0020037, FDR-adjusted *P* = 0.035), and flavin adenine dinucleotide (FAD) binding (GO: 0071949, FDR-adjusted *P* = 0.03), among others (see supplementary file 2, Supplementary Material online). FAD is the coenzyme for flavoprotein oxidoreductase enzymes which can contain iron and are important for ROS production (Messner and Imlay 2002). Likewise, heme proteins contain iron and play a key role in oxidoreductase activity (Zhang et al. 2019).

**Fig. 3.**
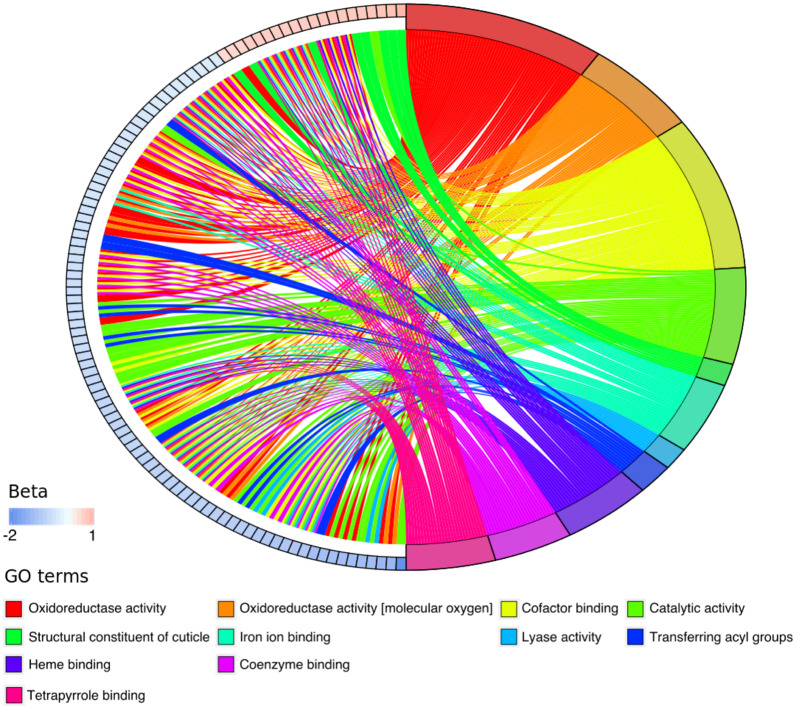
Coevolved *Enterococcus faecalis* caused the differential expression of 134 *Caenorhabditis elegans* host genes relative to evolved *E. faecalis* during infection by *Staphylococcus aureus*. The chord plot shows these genes along with the enriched molecular function gene ontology (GO)-terms (see Materials and Methods; supplementary files 1 and 2, Supplementary Material online). The differentially expressed genes are represented by boxes and ordered around the left half of the circle by beta values. Beta values are the effect size of differential expression; positive beta values indicate the upregulation of a gene, whereas negative values indicate downregulation. The molecular functions to which each gene links are depicted on the right half of the circle. For ease of visualization, we have collapsed molecular functions into broader “parental terms” (we set the minimum term size to 120 within g: profiler).

We have presented the 134 genes and the corresponding molecular functions as a chord plot in figure 3, where for ease of visualization, we have collapsed molecular functions into broader “parental terms” (we set the minimum term size to 120 within g: profiler). This plot shows that most genes, in particular those relating to oxidoreductase activity, have been relatively downregulated (depicted by beta values which are the natural log-fold change in expression). These changes could, however, lead to increased or decreased ROS production by nematodes due to the complex interaction of genes within and across pathways.

The same group of 134 genes that were differentially expressed in infected hosts responding to coevolved *E. faecalis* (relative to the evolved *E. faecalis* treatment), showed enrichment for cellular compartments linked to ROS production and iron-utilization. These cellular compartments include microbody (GO: 0042579, FDR-adjusted *P* = 0.0005), peroxisome (GO: 0005777, FDR-adjusted *P* = 0.0005; KEGG: 04146, FDR-adjusted *P* = 0.003), and mitochondrion (GO: 0005739, FDR-adjusted *P* = 0.005) (see supplementary file 2, Supplementary Material online). These compartments are sites of iron utilization and can generate ROS. The mitochondrion synthesizes iron-containing prosthetic groups including heme and iron–sulfur clusters and is the site of the electron transport chain which produces ROS. In addition, peroxisomes are microbodies that also contain catalase enzymes which contain iron and remove hydrogen peroxide.

Taken together, these results suggest that coevolved and evolved *E. faecalis* populations are differentially affecting nematode iron and ROS homeostasis. ROS production is a major protective mechanism by *E. faecalis*, but also an effective antipathogen defense across numerous animal species including *C. elegans* (Chávez et al. 2007). These molecules can also cause damage to animal host cells (Staerck et al. 2017), suggesting that the cost–benefit trade-off for the host to harbor *E. faecalis* may be the result of antagonistic pleiotropy in the ROS mechanism. Moreover, iron homeostasis is inherently linked to ROS production. Too much cellular iron catalyzes the generation of ROS that damages DNA and proteins, whereas too little cellular iron causes cell cycle arrest and cell death (Anderson and Leibold 2014). We know from previous research that *S. aureus* produces siderophore proteins that bind iron (Ford et al. 2016). It is therefore likely that increased *S. aureus* suppression by coevolved *E. faecalis* alters iron availability relative to evolved *E. faecalis.* We therefore hypothesize that nematodes are making plastic physiological adjustments to respond to either *E. faecalis*-produced ROS, the reduction in *S. aureus* infection load, or both. These factors resulting from the microbial warfare within may reduce the incentive for hosts to produce ROS themselves.

### Increased Microbe-Mediated Protection Downregulates a Key Immune Gene in Infected Hosts

**Fig. 4.**
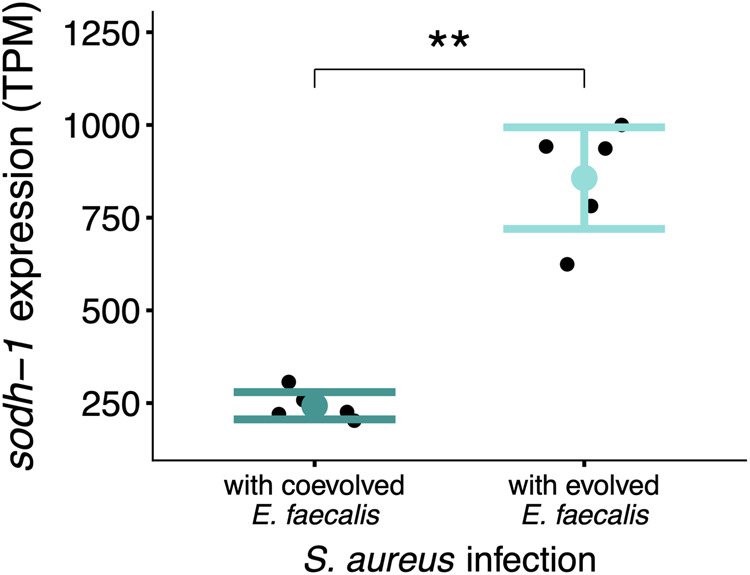
Coevolved *Enterococcus faecalis* downregulates the expression of in infected *Caenorhabditis elegans* compared with *E. faecalis* that evolved alone. After 12 h of exposure to *Staphylococcus aureus* and either *E. faecalis* from the coevolution or evolution treatment, we measured host transcription. Here, we show the transcripts per million (TPM) of the most significantly differentially expressed gene between the two treatments, (supplementary file 1, Supplementary Material online, Sleuth test for differential expression: , adjusted *P* = 0.0033, test statistic= 27.7, df = 1, beta value=−1.27). *P < 0.05, **P < 0.01, ***P < 0.001. Bar plots show ±2SE.

Of the 134 differentially regulated genes, the most significantly differentiated was a key immune gene, *.* This gene encodes a sorbitol dehydrogenase that has been shown to be upregulated in *C. elegans* upon *S. aureus* infection and to contribute toward nematode resistance to this pathogen (Irazoqui et al. 2010). Upregulation of has also been documented in nematodes infected by other pathogens (O’Rourke et al. 2006). We found that was significantly downregulated in nematodes colonized by coevolved *E. faecalis* compared with evolved *E. faecalis* populations (supplementary file 1, Supplementary Material online, fig. 4, Sleuth test for differential expression: adjusted *P* = 0.0033, test statistic= 27.7, df = 1, beta value=−1.27). This downregulation was only observed in *S. aureus*-infected hosts (we did not find any differentially expressed genes when comparing hosts monocolonized by protective microbes, see previous section). These findings indicate that stronger microbe-mediated protection against pathogens causes a plastic reduction in a key host immune gene against *S. aureus.* Our results do not however rule out the involvement of other host immune genes in defense or that might be upregulated at other time points to compensate for the downregulation of we observed. Moreover, future work should measure the protein activity to investigate functional links with immune system responses.

There may be a relationship between , oxidative stress, and antipathogen defense in our system. Electronic annotation (NCBI) infers that has oxidoreductase activity (GO: 0016491; GO: 0055114), suggesting a role in regulating host ROS levels. Previous research has also found that can be positively regulated by the DAF-16-mediated stress response pathway in *C. elegans* (Senchuk et al. 2018) with *daf-16* expression itself positively regulated by host ROS. Conversely, we find that the protective *E. faecalis* populations producing more ROS drove relatively lower expression during host infection. Given we also do not see differential regulation of *daf-16*, could be controlled by more than one pathway, or the relationship between ROS and is more complex. We do note a slight downregulation of the gene *lbp-5* in the coevolution treatment, a gene which can regulate expression (Xu et al. 2014). However, contrary to our findings, a deficiency in *lbp-5* expression has been shown to decrease oxidative stress resistance, and ramp up ROS production as well as expression by *C. elegans* (Xu et al. 2014). The extent to which interactions between these pathways control , and with expression-level dependent regulation, is unclear. Nonetheless, the apparent relationship between , oxidative stress, and defense against pathogens warrants further research in protective microbiota research.

### Conclusion

Microorganisms can rapidly evolve in response to the ecological interactions taking place within the host microbiome (Barroso-Batista et al. 2020), but also with infecting pathogens (Ford et al. 2017). We found that the coevolutionary race between protective and pathogenic microbial species in animal populations caused a trade-off whereby enhanced microbe-mediated defense was associated with a larger cost. Specifically, we found that in vivo coevolution with *S. aureus* favored *E. faecalis* populations that produce more ROS. Patterns of host transcription indicated that hosts responded to increases in the strength of microbe-mediated protection. Highly protected hosts differentially regulated iron metabolism and ROS production pathways, suggesting hosts monitored changes in their internal environment caused by the ROS protective microbial weaponry. These hosts may also have divested of a component of their own defenses in favor of the microbial one. They reduced expression of an important immune gene, , known to be useful in *C. elegans* against *S. aureus* and other pathogens. These findings hint at the ability of hosts to maintain control over their microbiota by detecting and responding to evolutionary changes in beneficial as well as harmful microbial traits (Foster et al. 2017).

The dynamic balance between the evolving benefits and costs highlights the riskiness of a host strategy to depend on protective microbiota for antipathogen defenses. Once the pathogen is cleared or absent from the community, the microbial symbiont may become too expensive. As infection risks vary during the lifetime of a larger host, maintaining plasticity in immune responses may allow hosts to break up the symbiosis when costs are too great (Palmer et al. 2008). Evolved dependence may make hosts highly vulnerable to harm from protective microbes, but also to pathogens if the microbial line of defense was ever lost. The evolution of host dependence on microbes is uncommon among defensive symbioses in nature, compared with nutritional host–microbe symbioses (Fisher et al. 2017), perhaps because of these shifts in the net benefits.

## Materials and Methods

### Nematode Host and Bacteria System

The simultaneous hermaphroditic N2 wild-type *C. elegans* was sourced from the CGC (University of Minnesota, MN). A genetically homogenous line was generated by selfing a single hermaphrodite for five generations. Populations of these worms were frozen in 50% M9 solution and 50% liquid freezing solution in cryotubes at −80 °C (Hope 1999). Populations were regularly resurrected throughout experimentation to prevent the accumulation of de novo mutations in host populations. Nematodes were maintained at 20 °C on nematode growth medium (NGM) with *Escherichia coli* OP50, a standard lab-based food (Hope 1999). The *E. coli* OP50 was grown at 30 °C shaking (200 rpm) overnight in lysogeny broth and 100 µl of culture was spread on NGM plates and incubated overnight at 30 °C (Hope 1999). To clean stocks and synchronize life stages, worms were treated with NaClO and NaOH which kills everything except unhatched worm eggs (Hope 1999).

We used *S. aureus* MSSA 476 (GenBank accession number BX571857.1), an invasive community-acquired methicillin-susceptible isolate, as the pathogen in our system. As the protective microbe, we used *Enterococcus faecalis* OG1RF (GenBank accession number CP002621.1), an isolate from the human digestive tract. A single ancestral population of each species was grown from a single colony overnight in 6 ml Todd Hewitt Broth (THB) shaking at 200 rpm at 30 °C. Bacteria were frozen in a 1:1 ratio of sample to 50% glycerol solution in cryotubes at −80 °C.

### In Vivo Experimental Coevolution

The evolution experiment consisted of two treatments: 1) *S. aureus* and *E. faecalis* were copassaged within *C. elegans* (and so allowed to coevolve), and 2) *E. faecalis* was passaged alone within *C. elegans* (fig. 1*A*). Each treatment consisted of five replicate populations with ten passages. We have previously found that this number of passages is sufficient for microbial evolution in the traits of interest to occur within populations of *C. elegans* (King et al. 2016).

Populations of *E. faecalis* and *S. aureus* were cultured overnight in 6 ml THB shaking (200 rpm) at 30 °C. After standardizing the cultures to OD600 of 1.00, 120 µl *E. faecalis* liquid culture or a mixture of 120 µl *S. aureus* and 120 µl *E. faecalis* was spread onto Trypic Soy Broth (TSB) agar plates and grown overnight at 30 °C. Both treatments provided bacterial cells in abundance to avoid differences in cell numbers ingested by the worms. Approximately 1,000 young adult nematodes were exposed to each replicate plate across both treatments at 25 °C. This was done by homogenously mixing a solution of worms in M9 buffer and using a pipette to measure how many worms were in 5 µl droplets. We found that the most accurate results were achieved by cutting the end of the pipette tip for a wider aperture. We took five of these measurements and calculated an average. We then calculated how much of the worm-M9 solution to add to each plate in order to achieve ∼1,000 worms. We allowed the M9 on the exposure plates to dry for 10 min prior to incubation.

After 24-h exposure, ten dead worms per replicate (considered dead after not responding to touch with a platinum wire) were washed by being transferred with platinum wire between five 5-µl drops of M9 buffer. Worms were then crushed in 20 µl M9 with a pestle and streaked onto selective media (TSB with 100 µg/ml rifampicin to select *E. faecalis* and Mannitol Salt Agar, MSA to select *S. aureus*) and cultured overnight at 30 °C. Ten colonies per species per replicate were grown in THB overnight, shaking (200 rpm) at 30 °C, and then used to make the exposure plates for the next passage. The above steps were repeated nine more times.

### Evolution of Microbe-Mediated Protection and Costs to Hosts

Protective ability was assessed by calculating the proportion of dead worms in the population after 24-h pathogen exposure, with and without *E. faecalis* cocolonization. Cost was assessed by calculating the proportion of dead worms in the population after 24-h exposure to *E. faecalis.* The protocol of exposure was the same as in the coevolution experiment (see above). Approximately 150 young adult worms from the stock population were placed onto exposure plates and incubated at 25 °C for 24 h. Exposure plates were labeled with a random code so that the treatments were unknown during measurement. The total number of worms and the number of dead worms were counted and the proportion dead calculated as mortality rate. Both cost and protection experiments were repeated twice independently and the counts of total and dead worms were summed prior to statistical analysis.

### Reactive Oxygen Species Production

*Enterococcus faecalis*-mediated suppression of *S. aureus* via ROS production was assessed in vitro. We performed this experiment with either coevolved or evolved populations of *E. faecalis* against the ancestral stock of *S. aureus.* Bacteria were cultured overnight in THB shaking (200 rpm) at 30 °C. THB solution was made with 0.25 M potassium phosphate buffer containing SOD from bovine erythrocytes (Sigma-Aldrich) and CAT from bovine liver (Sigma-Aldrich), each at a concentration of 0.25 mg ml^−1^. An enzyme-free THB solution served as control with only 0.25 M potassium phosphate buffer. After standardizing the bacteria (OD600 of 1.00), 3 µl of each species was added to 194 µl of THB and shaken at 30 °C for 24 h. Colony-forming units per ml (CFU/ml) of *S. aureus* were counted by plating dilutions onto MSA plates. This experiment was repeated two independent times and the results were averaged per replicate population.

### Host RNA Extraction and Analysis

We examined host gene expression upon sole colonization with *E. faecalis* populations that either coevolved with *S. aureus* compared with *E. faecalis* populations that evolved on their own in the host (fig. 2*B*). We subsequently measured how these protective microbes affected host gene expression in response to infection by *S. aureus* by performing cocolonization experiments (fig. 2*B*).

Sterile and age-synchronized nematode eggs were collected using the bleach-sodium hydroxide solution. These eggs were kept in M9 buffer without food, shaking overnight at 88 rpm and 20 °C to further synchronize the age of each worm. These worms were then transferred to NGM plates with *E. coli* as food and kept at 20 °C. Approximately 5,000 worms were raised on each plate for 2 days. *Enterococcus faecalis* and *S. aureus* were then grown overnight in 6 ml THB in a shaking incubator (200 rpm) at 30 °C. Exposure plates were made in the same way as for the evolution experiment (see above). At this point, worms were washed three times in 50 ml M9 buffer using gravity. Approximately 2,000 young adult worms were then exposed to the bacterial lawns and were incubated at 25 °C for 12 h.

After 12 h of exposure to the bacterial treatments, we collected the worms in M9 buffer and washed them five times by diluting them in 10 ml M9 buffer, using gravity to settle them between washes. Populations were processed randomly across treatments (using a random number generator) and within 10 min of collection. For each replicate, ∼1,000 worms were collected from each sample using the technique described above (see In Vivo Experimental Coevolution) and placed into an Eppendorf tube containing 1 ml M9 buffer. When the worms settled at the bottom of this tube, we used a pipette set to 50 µl to collect the pellet of ∼1,000 worms in 50 µl M9 buffer. We then added this 50 µl M9 solution of worms to 1 ml of Trizol in an Eppendorf tube. This worm collection protocol ensured a consistent concentration and number of worms over all samples. Samples were vortexed for 20 s, freeze-thawed three times to break the worm cuticle using dry ice and a heat block (40 °C), and stored at −80 °C. RNA was extracted using Zymo spin columns with on-column DNA digestion using DNase I. RNA was quantified by Qubit (Invitrogen) and all samples diluted to the same final concentration. Library preparation and sequencing were performed (Oxford Genomics Centre). The mRNA fraction was selected from the RNA using the polyA signal and converted to cDNA. Second-strand cDNA synthesis incorporated dUTP. The cDNA was then end-repaired, A-tailed, and adapter-ligated. Prior to amplification, samples underwent uridine digestion. The prepared libraries were size selected, multiplexed, and quality controlled before paired-end sequencing over five units of a flow cell. Sequencing was carried out using NovaSeq6000 with 150-bp paired-end reads.

Raw reads were checked for quality using FastQC (0.11.5). Release 96 GTF and cDNA FASTA files were downloaded from the ensemble database for *C. elegans* (WBcel235) and a transcript index was created using *kallisto*. Pseudoalignment was performed using *kallisto* with 100 bootstraps.

### Statistical Analysis

Pseudoalignment outputs from *kallisto* were analyzed using *sleuth* in R v 3.2.0 (http://www.r-project.org/), testing for differential gene expression by treatment and correcting for multiple comparisons. GO term enrichment analysis on the target IDs of differentially expressed genes used the g: Profiler tool online with the Benjamini and Hochberg false discovery rate (FDR) correction for multiple comparisons (Raudvere et al. 2019). The remaining analyses were performed within R. The Shapiro test was used to detect whether data were normally distributed and *F*-tests to compare the variances of two samples from normal populations. A binomial GLM was used to compare *E. faecalis* cost, based on the number of live and dead worms. To account for overdispersion, a quasibinomial GLM was used to compare *E. faecalis* protective ability, based on the number of live and dead worms. Plots of the GLMs were checked by eye for model quality and Tukey contrasts were used for post hoc comparisons. *t*-Tests were used to compare *E. faecalis* suppression of *S. aureus* via ROS and *P* values were corrected using the p.adjust() function with the Benjamini and Hochberg FDR method. 

## Supplementary Material

Supplementary data are available at *Molecular Biology and Evolution* online.

## Supplementary Material

msaa292_Supplementary_Data

## References

[msaa292-B1] Anderson CP , Leibold EA. 2014. Mechanisms of iron metabolism in *Caenorhabditis elegans*. Front Pharmacol. 5:113. 24904417 10.3389/fphar.2014.00113 PMC4033076

[msaa292-B2] Ashby B , King KC. 2017. Friendly foes: the evolution of host protection by a parasite. Evol Lett. 1(4):211–221. 30283650 10.1002/evl3.19 PMC6121858

[msaa292-B3] Barroso-Batista J , Pedro MF, Sales-Dias J, Pinto CJG, Thompson JA, Pereira H, Demengeot J, Gordo I, Xavier KB. 2020. Specific eco-evolutionary contexts in the mouse gut reveal *Escherichia coli* metabolic versatility. Curr Biol. 30(6):1049–1062. 32142697 10.1016/j.cub.2020.01.050

[msaa292-B4] Cayetano L , Rothacher L, Simon JC, Vorburger C. 2015. Cheaper is not always worse: strongly protective isolates of a defensive symbiont are less costly to the aphid host. Proc R Soc B. 282(1799):20142333. 10.1098/rspb.2014.2333 PMC4286048 25473015

[msaa292-B5] Chávez V , Mohri-Shiomi A, Maadani A, Vega LA, Garsin D. 2007. Oxidative stress enzymes are required for DAF-16-mediated immunity due to generation of reactive oxygen species by *Caenorhabditis elegans*. Genetics 176(3):1567–1577. 17483415 10.1534/genetics.107.072587 PMC1931534

[msaa292-B6] Clark LC , Hodgkin J. 2014. Commensals, probiotics and pathogens in the *Caenorhabditis elegans* model. Cell Microbiol. 16(1):27–38. 24168639 10.1111/cmi.12234

[msaa292-B7] Degnan PH , Moran NA. 2008. Diverse phage-encoded toxins in a protective insect endosymbiont. Appl Environ Microbiol. 74(21):6782–6791. 18791000 10.1128/AEM.01285-08 PMC2576707

[msaa292-B8] Fisher RM , Henry LM, Cornwallis CK, Kiers ET, West SA. 2017. The evolution of host-symbiont dependence. Nat Commun. 8(1):15973. 28675159 10.1038/ncomms15973 PMC5500886

[msaa292-B17785642] Ford SA King KC. 2020. Data from: In vivo microbial coevolution favors host protection and plastic downregulation of immunity, Dryad, Dataset. Available from: 10.5061/dryad.3xsj3txcj. 10.1093/molbev/msaa292 PMC8042738 33179739

[msaa292-B9] Ford SA , Kao D, Williams D, King KC. 2016. Microbe-mediated host defence drives the evolution of reduced pathogen virulence. Nat Commun. 7(1):13430. 27845328 10.1038/ncomms13430 PMC5116080

[msaa292-B10] Ford SA , King KC. 2016. Harnessing the power of defensive microbes: evolutionary implications in nature and disease control. PLoS Pathog. 12(4):e1005465. 27058881 10.1371/journal.ppat.1005465 PMC4826280

[msaa292-B11] Ford SA , Williams D, Paterson S, King KC. 2017. Co-evolutionary dynamics between a defensive microbe and a pathogen driven by fluctuating selection. Mol Ecol. 26(7):1778–1789. 27862515 10.1111/mec.13906 PMC6849518

[msaa292-B12] Foster KR , Schluter J, Coyte KZ, Rakoff-Nahoum S. 2017. The evolution of the host microbiome as an ecosystem on a leash. Nature 548(7665):43–51. 28770836 10.1038/nature23292 PMC5749636

[msaa292-B13] Frank SA. 1996. Models of parasite virulence. Q Rev Biol. 71(1):37–78. 8919665 10.1086/419267

[msaa292-B14] Gerardo NM , Altincicek B, Anselme C, Atamian H, Barribeau SM, de Vos M, Duncan EJ, Evans JD, Gabaldón T, Ghanim M, et al 2010. Immunity and other defenses in pea aphids, *Acyrthosiphon pisum*. Genome Biol. 11(2):R21. 20178569 10.1186/gb-2010-11-2-r21 PMC2872881

[msaa292-B15] Granato ET , Meiller-Legrand TA, Foster KR. 2019. The evolution and ecology of bacterial warfare. Curr Biol. 29(11):R521–R537. 31163166 10.1016/j.cub.2019.04.024

[msaa292-B16] Hall JPJ , Brockhurst MA, Harrison E. 2017. Sampling the mobile gene pool: innovation via horizontal gene transfer in bacteria. Philos Trans R Soc Lond B Biol Sci. 372(1735):20160424. 10.1098/rstb.2016.0424 PMC5665811 29061896

[msaa292-B17] Heikkilä MP , Saris PE. 2003. Inhibition of *Staphylococcus aureus* by the commensal bacteria of human milk. J Appl Microbiol. 95(3):471–478. 12911694 10.1046/j.1365-2672.2003.02002.x

[msaa292-B18] Hope IA. 1999. *C. elegans*: a practical approach. Oxford: Oxford University Press.

[msaa292-B19] Huycke MM , Abrams V, Moore DR. 2002. *Enterococcus faecalis* produces extracellular superoxide and hydrogen peroxide that damages colonic epithelial cell DNA. Carcinogenesis 23(3):529–536. 11895869 10.1093/carcin/23.3.529

[msaa292-B20] Irazoqui JE , Troemel ER, Feinbaum RL, Luhachack LG, Cezairliyan BO, Ausubel FM. 2010. Distinct pathogenesis and host responses during infection of *C. elegans* by *P. aeruginosa* and *S. aureus*. PLoS Pathog. 6(7):e1000982. 20617181 10.1371/journal.ppat.1000982 PMC2895663

[msaa292-B21] Kaltenpoth M , Engl T. 2014. Defensive microbial symbionts in Hymenoptera. Funct Ecol. 28(2):315–327.

[msaa292-B22] King KC , Brockhurst MA, Vasieva O, Paterson S, Betts A, Ford SA, Frost CL, Horsburgh MJ, Haldenby S, Hurst GDD. 2016. Rapid evolution of microbe-mediated protection against pathogens in a worm host. ISME J. 10(8):1915–1924. 26978164 10.1038/ismej.2015.259 PMC5029159

[msaa292-B23] Koch H , Schmid-Hempel P. 2012. Gut microbiota instead of host genotype drive the specificity in the interaction of a natural host-parasite system. Ecol Lett. 15(10):1095–1103. 22765311 10.1111/j.1461-0248.2012.01831.x

[msaa292-B24] Kroiss J , Kaltenpoth M, Schneider B, Schwinger MG, Hertweck C, Maddula RK, Strohm E, Svatos A. 2010. Symbiotic Streptomycetes provide antibiotic combination prophylaxis for wasp offspring. Nat Chem Biol. 6(4):261–263. 20190763 10.1038/nchembio.331

[msaa292-B25] Kwiatkowski M , Engelstädter J, Vorburger C. 2012. On genetic specificity in symbiont-mediated host-parasite coevolution. PLoS Comput Biol. 8(8):e1002633. 22956894 10.1371/journal.pcbi.1002633 PMC3431304

[msaa292-B26] Lindsey ARI , Bhattacharya T, Newton ILG, Hardy RW. 2018. Conflict in the intracellular lives of endosymbionts and viruses: a mechanistic look at *Wolbachia*-mediated pathogen-blocking. Viruses 10(4):141. 10.3390/v10040141 PMC5923435 29561780

[msaa292-B27] Martinez J , Cogni R, Cao C, Smith S, Illingworth CJ, Jiggins FM. 2016. Addicted? Reduced host resistance in populations with defensive symbionts. Proc Biol Sci. 283(1833):20160778. 10.1098/rspb.2016.0778 PMC4936038 27335421

[msaa292-B28] Martinez J , Ok S, Smith S, Snoeck K, Day JP, Jiggins FM. 2015. Should symbionts be nice or selfish? Antiviral effects of *Wolbachia* are costly but reproductive parasitism is not. PLoS Pathog. 11(7):e1005021. 26132467 10.1371/journal.ppat.1005021 PMC4488530

[msaa292-B29] Martín-Platero AM , Valdivia E, Ruíz-Rodríguez M, Soler JJ, Martín-Vivaldi M, Maqueda M, Martínez-Bueno M. 2006. Characterization of antimicrobial substances produced by *Enterococcus faecalis* MRR 10-3, isolated from the uropygial gland of the hoopoe (*Upupa epops*). Appl Environ Microbiol. 72(6):4245–4249. 16751538 10.1128/AEM.02940-05 PMC1489579

[msaa292-B30] Mejía LC , Herre EA, Sparks JP, Winter K, García MN, Van Bael SA, Stitt J, Shi Z, Zhang Y, Messner KR, et al 2002. Mechanism of superoxide and hydrogen peroxide formation by fumarate reductase, succinate dehydrogenase, and aspartate oxidase. J Biol Chem. 277(45):42563–42571. 12200425 10.1074/jbc.M204958200

[msaa292-B8811001] Messner KR, Imlay JA. 2002. Mechanism of superoxide and hydrogen peroxide formation by fumarate reductase, succinate dehydrogenase, and aspartate oxidase. *J Biol Chem.* 277(45):42563–42571. 12200425 10.1074/jbc.M204958200

[msaa292-B31] Metcalf CJE , Koskella B. 2019. Protective microbiomes can limit the evolution of host pathogen defense. Evol Lett. 3(5):534–543. 31636945 10.1002/evl3.140 PMC6791398

[msaa292-B32] Montalvo-Katz S , Huang H, Appel MD, Berg M, Shapira M. 2013. Association with soil bacteria enhances p38-dependent infection resistance in *Caenorhabditis elegans*. Infect Immun. 81(2):514–520. 23230286 10.1128/IAI.00653-12 PMC3553824

[msaa292-B33] Nelson PG , May G. 2020. Defensive symbiosis and the evolution of virulence. Am Nat. 196(3):333–343. 32813997 10.1086/709962

[msaa292-B34] O’Rourke D , Baban D, Demidova M, Mott R, Hodgkin J. 2006. Genomic clusters, putative pathogen recognition molecules, and antimicrobial genes are induced by infection of *C. elegans* with *M. nematophilum*. Genome Res. 16(8):1005–1016. 16809667 10.1101/gr.50823006 PMC1524860

[msaa292-B35] Oliver KM , Campos J, Moran NA, Hunter MS. 2008. Population dynamics of defensive symbionts in aphids. Proc R Soc B. 275(1632):293–299. 10.1098/rspb.2007.1192 PMC2593717 18029301

[msaa292-B36] Oliver KM , Smith AH, Russel JA. 2014. Defensive symbiosis in the real world – advancing ecological studies of heritable, protective bacteria in aphids and beyond. Funct Ecol. 28(2):341–355.

[msaa292-B37] Palmer TM , Stanton ML, Young TP, Goheen JR, Pringle RM, Karban R. 2008. Breakdown of an ant-plant mutualism follows the loss of large herbivores from an African savanna. Science 319(5860):192–195. 18187652 10.1126/science.1151579

[msaa292-B38] Pan X , Zhou G, Wu J, Bian G, Lu P, Raikhel AS, Xi Z. 2012. *Wolbachia* induces reactive oxygen species (ROS)-dependent activation of the Toll pathway to control dengue virus in the mosquito *Aedes aegy*pti. Proc Natl Acad Sci U S A. 109(1):E23–E31. 22123956 10.1073/pnas.1116932108 PMC3252928

[msaa292-B39] Parker BJ , Barribeau SM, Laughton AM, de Roode JC, Gerardo NM. 2011. Non-immunological defense in an evolutionary framework. Trends Ecol Evol. 26(5):242–248. 21435735 10.1016/j.tree.2011.02.005

[msaa292-B40] Petersen C , Dirksen P, Schulenburg H. 2015. Why we need more ecology for genetic models such as *C. elegans*. Trends Genet. 31(3):120–127. 25577479 10.1016/j.tig.2014.12.001

[msaa292-B41] Polin S , Simon JC, Outreman Y. 2014. An ecological cost associated with protective symbionts of aphids. Ecol Evol. 4(6):836–830. 10.1002/ece3.991 PMC3967907 24683464

[msaa292-B42] Raudvere U , Kolberg L, Kuzmin I, Arak T, Adler P, Peterson H, Vilo J. 2019. g: profiler: a web server for functional enrichment analysis and conversions of gene lists (2019 update). Nucleic Acids Res. 47(W1):W191–W198. 31066453 10.1093/nar/gkz369 PMC6602461

[msaa292-B43] Rossouw W , Korsten L. 2017. Cultivable microbiome of fresh white button mushrooms. Lett Appl Microbiol. 64(2):164–170. 27930823 10.1111/lam.12698

[msaa292-B44] Rouchet R , Vorburger C. 2012. Strong specificity in the interaction between parasitoids and symbiont-protected hosts. J Evol Biol. 25(11):2369–2375. 22998667 10.1111/j.1420-9101.2012.02608.x

[msaa292-B45] Senchuk MM , Dues DJ, Schaar CE, Johnson BK, Madaj ZB, Bowman MJ, Winn ME, Van Raamsdonk JM. 2018. Activation of DAF-16/FOXO by reactive oxygen species contributes to longevity in long-lived mitochondrial mutants in *Caenorhabditis elegans*. PLoS Genet. 14(3):e1007268. 29522556 10.1371/journal.pgen.1007268 PMC5862515

[msaa292-B46] Staerck C , Gastebois A, Vandeputte P, Calenda A, Larcher G, Gillmann L, Papon N, Bouchara JP, Fleury MJJ. 2017. Microbial antioxidant defense enzymes. Microb Pathog. 110:56–65. 28629723 10.1016/j.micpath.2017.06.015

[msaa292-B47] Vorburger C , Perlman SJ. 2018. The role of defensive symbionts in host-parasite coevolution. Biol Rev. 93(4):1747–1764. 29663622 10.1111/brv.12417

[msaa292-B48] Weiss BL , Maltz M, Aksoy S. 2012. Obligate symbionts activate immune system development in the tsetse fly. J Immunol. 188(7):3395–3403. 22368278 10.4049/jimmunol.1103691 PMC3311772

[msaa292-B49] West SA , Buckling A. 2003. Cooperation, virulence and siderophore production in bacterial parasites. Proc R Soc Lond B. 270(1510):37–44. 10.1098/rspb.2002.2209 PMC1691207 12590769

[msaa292-B50] Xu M , Choi EY, Paik YK. 2014. Mutation of the *lbp-5* gene alters metabolic output in *Caenorhabditis elegans*. BMB Rep. 47(1):15–20. 24195791 10.5483/BMBRep.2014.47.1.086 PMC4163843

[msaa292-B51] Zhang J , Li X, Olmedo M, Holdorf AD, Shang Y, Artal-Sanz M, Yilmaz LS, Walhout AJM. 2019. A delicate balance between bacterial iron and reactive oxygen species supports optimal *C. elegans* development. Cell Host Microbe. 26(3):400–411.e3. 31444089 10.1016/j.chom.2019.07.010 PMC6742550

