# Supplementary material for: In Vivo Microbial Coevolution Favors Host Protection and Plastic Downregulation of Immunity
Source: Mol Biol Evol. 2020 Nov 12;38(4):1330–8. doi: 10.1093/molbev/msaa292 (PMC8042738; doi:10.1093/molbev/msaa292)
Supplement: msaa292_Supplementary_Data [file msaa292_supplementary_data.zip › Supplementary Table 1.docx]

**Supplementary Table 1.** All mutations found in *E. faecalis* listed by evolutionary treatment and replicate population^a^.

| Treatment  Supplementary Table 1. Coevolution S aureus. SNPS >20% frequency | Replicate | Locus tag/  coordinate | Mutation | % SNP abundance | Gene name/annotation | Putative function | Putative link with  ROS if present | Reference  (PMID) |
| --- | --- | --- | --- | --- | --- | --- | --- | --- |
| **Coevolution** | 1 | OG1RF_RS03810  OG1RF_10736 | N | 68 | isoleucine--tRNA ligase | Catalyzes the attachment of isoleucine to tRNA(Ile) |  |  |
|  | 2 | 1787496 |  | 25 | Between hypothetical protein and MOP flippase transporter OG1RF_11709 | MOP flippase transporter is important for cell surface biology |  |  |
|  |  | OG1RF_RS1193  OG1RF_123290 | S | 18 | Protein-tyrosine-phosphatase (PTP) | Controls tyrosine phosphorylation for cell signalling | Oxidation of PTPs has been linked to response to ROS | 21856739 |
|  | 3 | OG1RF_RS03840  OG1RF_10742 | N | 43 | DEAD/DEAH box family ATP-dependent RNA helicase | RNA helicases associated with cellular metabolism | Mitochondrial generation of ROS in plants | 18710941 22652060 |
|  |  | 2603433 |  | 18 | Between OsmC/Ohr family protein and 30S ribosomal protein S14 | OsmC/Ohr is a peroxiredoxin-expressed in response to oxidative stress | Scavenge ROS | 28634401 |
|  | 4 | OG1RF_RS09200  OG1RF_11797 | */ | 58 | Xanthine permease | Transporter for xanthine | Xanthine can be oxidised to produce ROS | 11895869 |
|  |  | OG1RF_RS00060  OG1RF_10010 | N | 20 | DHH family phosphoesterase | Nucleic acid binding |  |  |
|  |  | OG1RF_RS07935  OG1RF_11546 | * | 20 | PurE, phosphoribosylaminoimidazole carboxylase catalytic subunit | The chemical reactions and pathways resulting in the formation of IMP, inosine monophosphate, | IMP is used in the Salvage pathway to produce Xanthine. Xanthine can be oxidised to produce ROS | 11895869 |
|  | 5 | 2033091 |  | 15 | Between hypothetical protein and cell wall surface anchor family protein |  |  |  |
| **Single Evolution** | 1 | OG1RF_RS09430  OG1RF_11842 | S | 100 | GTPase Era | Cell cycle regulation, energy metabolism | Energy metabolism |  |
|  | 2 | 1317832 |  | 93 | Between ribosomal large subunit pseudouridine synthase B (OG1RF_11259) and flavin mononuvleotide | flavin mononuvleotide functions as prosthetic group of various oxidoreductases | Flavin-containing oxidoreductases are those that are known to lead to ROS production | 12200425 |
|  | 3 | OG1RF_RS09105  OG1RF_11779 | S | 63 | hypothetical protein |  |  |  |
|  |  | OG1RF_RS08605  OG1RF_11678 | N | 20 | ABC superfamily ATP binding cassette transporter,  membrane protein |  |  |  |
|  | 4 | No mutations ≥15% |  |  |  |  |  |  |
|  | 5 | 1317832 |  | 48 | Between ribosomal large subunit pseudouridine synthase B (OG1RF_11259) and flavin mononuvleotide | flavin mononuvleotide functions as prosthetic group of various oxidoreductases | Flavin-containing oxidoreductases are those that are known to lead to ROS production | 12200425 |

**^a^Supplementary Table 1**

All mutations found in *E. faecalis* listed by evolutionary treatment and replicate population. Locus tag is given when mutation occurs in a coding region. Otherwise, the coordinate is given. Mutation abundance is the percentage of 40 sampled clones that contain the mutation in the replicate population. Only mutations present in >15% clones are listed to control false positives at the expense of low frequency true positives. Gene names and putative gene function are given where known, along with a reference. A proposed link with ROS is listed where identified. Mutation type: S=synonymous. N=non-synonymous. *=stop gain. */= stop loss.
